# Bimetallic zeolite-imidazole framework-based heterostructure with enhanced photocatalytic hydrogen production activity†

**DOI:** 10.1039/d1ra00781e

**Published:** 2021-03-01

**Authors:** Nayab Arif, Ye-Zhan Lin, Kai Wang, Yi-Chuan Dou, Yu Zhang, Kui Li, Shiquan Liu, Fu-Tian Liu

**Affiliations:** School of Materials Science and Engineering, University of Jinan Jinan 250022 China mse_lik@ujn.edu.cn vctrliu@hotmail.com

## Abstract

Bimetallic zeolite-imidazole frameworks with controllable flat band position, band gap and hydrogen evolution reaction characteristics were adopted as a photocatalytic hydrogen production catalyst. Furthermore, the g-C_3_N_4_–MoS_2_ 2D–2D surface heterostructure was introduced to the ZnM-ZIF to facilitate the separation as well as utilization efficiency of the photo-exited charge carriers in the ZnM-ZIFs. On the other hand, the ZnM-ZIFs not only inhibited the aggregation of the g-C_3_N_4_–MoS_2_ heterostructure, but also improved the separation and transport efficiency of charge carriers in g-C_3_N_4_–MoS_2_. Consequently, the optimal g-C_3_N_4_–MoS_2_–ZnNi-ZIF exhibited an extraordinary photocatalytic hydrogen evolution activity 214.4, 37.5, and 3.7 times larger than that of the pristine g-C_3_N_4_, g-C_3_N_4_–ZnNi-ZIF and g-C_3_N_4_–MoS_2_, respectively, and exhibited a H_2_-evolution performance of 77.8 μmol h^−1^ g^−1^ under UV-Vis light irradiation coupled with oxidation of H_2_O into H_2_O_2_. This work will furnish a new MOF candidate for photocatalysis and provide insight into better utilization of porous MOF-based heterostructures for hydrogen production from pure water.

## Abstract 2

The g-C_3_N_4_-MoS_2_ could facilitate the separation as well as utilization efficiency of the photo-generated charge carriers in the ZnM-ZIFs, and hence improved the photocatalytic H_2_ production with and without sacrificial agent.

## Graphical abstract

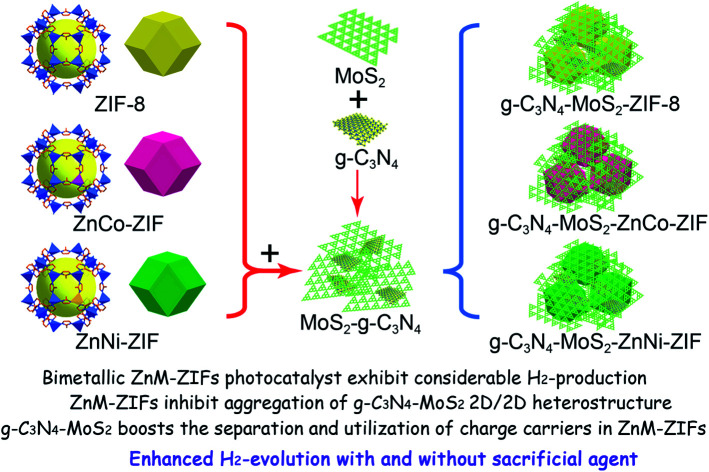

## Introduction

The direct conversion of sustainable solar power into eco-friendly energy over high performance photocatalysts has been extremely significant for powering human society.^1–7^ Up to now, it is universally recognized that the serious recombination, underutilization and poor transport of photon-generated carriers weaken the photocatalytic activity of the photocatalyst.^8–12^ Consequently, extensive efforts have been devoted to constructing heterostructures or phase junctions, which could increase the specific surface area of the photocatalyst,^2,13–21^ and adopting a suitable cocatalyst for improving the separation, transport and utilization of the photo-induced charge carriers.^10,11,22,23^ However, most of the photocatalyst systems were composed of inorganic metal oxides/sulfides, which may result in poor transport and separation efficiency of charge carriers, low porosity, and limited flexibility in material design. In this instance, metal–organic frameworks (MOFs), constructed from metal ions/clusters and organic linkers, exhibited various attractive characteristics such as high specific surface area, crystalline nature,^24–26^ and especially structure diversity for easy tailorability of the functionality,^24,27–29^ and have attracted widespread interest in photocatalytic applications. Hence, various Zr, Ti, Cr-based MOFs with and without Pt, small molecular and even polyoxometalates (POMs) have been extensively investigated as photocatalyst for hydrogen production.^25,26,30,31^

As one of the most stable metal organic frameworks in neutral and alkaline solutions,^26,32,33^ Zeolite Imidazole Frameworks (ZIFs) and its heterostructure has been extensively studied for the photocatalytic reaction.^33,34^ Especially, the bimetallic ZIFs has been widely investigated because it possesses various structures, has versatile functions and its photo-electric properties. Moreover, the bimetallic ZIF derived nanomaterials have been fabricated and used in the electrocatalytic energy conversion and storage.^25,32,34–36^ However, the bimetallic ZIFs itself with special merits for photocatalysis was rarely reported as photocatalyst, although this is a stirring work, and will bring out some new interesting prospects. Moreover, almost all the MOFs photocatalysts suffered from the problems of limited photocatalytic hydrogen production activity and poor photocatalytic stability even in sacrificial agent solution owing to the low photo-response, rapid recombination of photo-exited electron–hole pairs. Construction of ZIFs-based heterostructure not only resolved the problems of low porosity of semiconductors, but also improved the separation and utilization of the photo-generated charge carriers.

Graphic carbon nitride (g-C_3_N_4_), which is composed of the earth abundant carbon and nitrogen atoms through strong covalent bonds, possesses enhanced stability in solutions over a wide pH range, and suitable for the visible light absorption.^37–40^ The separation and utilization efficiency of the photo-generated charge carriers in g-C_3_N_4_ is very limited, resulting in poor photocatalytic activity.^9,23,41^ Consequently, a large number of cocatalysts were fabricated, and one of the most extensively investigated cocatalyst is MoS_2_.^9,42–45^ Due to the different work function of MoS_2_ compared with the semiconductor components, and its low hydrogen evolution reaction (HER) overpotential, the photo-exited electrons and holes in g-C_3_N_4_–MoS_2_ 2D–2D surface heterostructure could be transformed into different semiconductor components with high utilization efficiency. Therefore g-C_3_N_4_–MoS_2_ heterostructure was adopted to incorporate on ZIFs-based heterostructure that not only resolved the problems of low porosity, but also improved the separation and utilization of the photo-generated charge carriers between g-C_3_N_4_–MoS_2_–ZnM-ZIF. Simultaneously, ZnM-ZIF could restrain the agglomeration of g-C_3_N_4_–MoS_2_ during the subsequent post annealing and photocatalytic reactions.^9,46,47^ However, during the fabrication process and/or the photocatalytic reaction, the g-C_3_N_4_–MoS_2_ nanosheet may aggregate occur and thus deteriorate its photocatalytic hydrogen production activity.^34,48^ Moreover, the photocatalytic hydrogen evolution in pure water for g-C_3_N_4_–MoS_2_ and especially MOFs photocatalyst is important and it also faces huge challenges.^49^

Herein, bimetallic ZIFs (ZnM-ZIF, M = Co, Ni) with controllable band were synthesized and adopted as the photocatalytic hydrogen production catalyst with a considerable photocatalytic HER activity. The g-C_3_N_4_–MoS_2_ nanorods were prepared by one-step hydrothermal method. Further the g-C_3_N_4_–MoS_2_–ZnM-ZIFs (M = Co, Ni) were prepared using the similar method with g-C_3_N_4_–MoS_2_ as precursor. The introduction of g-C_3_N_4_–MoS_2_ onto the surface of the ZnM-ZIF not only improved the utilization efficiency of photo-exited charge carriers by MoS_2_, but also inhibited the accumulation of g-C_3_N_4_–MoS_2_ heterostructure. Hence, the optimal g-C_3_N_4_–MoS_2_–ZnNi-ZIF (CNMZN) exhibited the extraordinary photocatalytic HER activity of 30.1 μmol h^−1^, being 214.4, 37.5, and 3.7 times larger than that of the pristine g-C_3_N_4_ (CN), g-C_3_N_4_–ZnNi-ZIF (CNZN) and g-C_3_N_4_–MoS_2_ (CNM), respectively. More importantly, even in pure water, the optimal ternary heterostructures exhibited considerable hydrogen production rate. This work provided innovation that can hopefully help in designing a novel high-performance MOF-based heterostructure photocatalyst for solar conversion.

## Experimental section

### Materials and general methods

C_3_N_3_(NH_2_)_3_, Zn(NO_3_)_2_·6H_2_O, Ni(NO_3_)_2_·6H_2_O, Co(NO_3_)_2_·6H_2_O, C_4_H_6_N_2_, H_3_P(Mo_3_O_10_)_4_·*x*H_2_O and CH_4_N_2_S (Shanghai Macklin Biochemical Technology Co., Ltd) are analytical grade and used as received without further purification. The powder X-ray diffraction (PXRD) patterns were recorded on a D/max 2500 VL/PC diffractometer (Japan) equipped with graphite monochromatized Cu Kα radiation (*λ* = 1.54060 Å). Corresponding work voltage and current is 40 kV and 100 mA, respectively. The transmission electron microscopy (TEM) and high-resolution TEM (HRTEM) images were recorded on JEOL-2100F apparatus at an accelerating voltage of 200 kV. The atomic structure of the g-C_3_N_4_–MoS_2_-MOF heterojunction was characterized using an ARM-200CF (JEOL, Tokyo, Japan) transmission electron microscope operated at 200 kV and equipped with double spherical aberration correctors. Surface morphologies of the phase junction materials were examined by a scanning electron microscope (SEM, JSM-7600F) at an acceleration voltage of 10 kV. The UV-Vis absorption and diffused reflectance spectra were recorded using a Cary 5000 UV-Vis spectrometer (Viarian, USA) with BaSO_4_ as a reflectance standard. Nitrogen adsorption–desorption isotherms were measured at 77 K on a Quantachrome Instruments Autosorb AS-6B. The pore size distributions were measured by the Barrett–Joyner–Halenda (BJH) method. Steady photoluminescence (PL) emission spectra were tested by a luminescence spectrophotometer (QM-400, PTI) with 350 nm excitation wavelength. The transient photocurrent responses experiments were carried out at room temperature under visible light irradiation using a conventional three-electrode system with a glassy carbon electrode (3 mm in diameter, sheet resistance 20–25 Ω per square) as the working electrode, a carbon rod as the auxiliary electrode, and an Ag/AgCl electrode as the reference electrode. All the samples were dispersed in deionized water with a concentration of 1.5 mg mL^−1^ and deposited on the glassy carbon electrode and dried under visible light irradiation. The electrocatalytic hydrogen evolution reaction was measured in 1 M KOH solution. EIS data were recorded using electrochemical (Bio-Logic SP-150) workstation in Na_2_SO_4_ (0.5 M) solution. The transient photocurrent responses measurements were performed with a CHI 660E electrochemical station (Shanghai Chenhua Co. Ltd, China) under irradiation in 0.5 M Na_2_SO_4_ solutions.

### Synthesis of g-C_3_N_4_

The pure g-C_3_N_4_ photocatalyst was prepared by a traditional thermal polymerization strategy. In a typical procedure, 5 g of C_3_N_3_(NH_2_)_3_ was placed in a crucible with a cover, and then it was annealed at 650 °C for 2 h in the argon. The final yellow powder was then collected.

### Synthesis of the g-C_3_N_4_–MoS_2_

The g-C_3_N_4_–MoS_2_ nanorods were prepared using H_3_P(Mo_3_O_10_)_4_·*x*H_2_O, CH_4_N_2_S as precursors by one-step hydrothermal method. In a typical procedure, 200 mg g-C_3_N_4_, suitable amount of H_3_P(Mo_3_O_10_)_4_·*x*H_2_O (with Mo content of 6 wt% (19 mg) in comparison with g-C_3_N_4_) and CH_4_N_2_S (the corresponding ratios of Mo/S equal to 1 : 2) were dissolved in 30 mL distilled water. The solutions were subsequently transferred into 50 mL Teflon-lined autoclave and maintained for 24 h at 160 °C. The final black products were rinsed three times with distilled water and ethanol respectively, and dried at 60 °C for overnight in a vacuum oven to evaporate the solvent.

### Synthesis of the ZnNi-MOF, ZnCo-MOF and ZIF-8

The ZnNi-MOF nanorods were prepared using Zn(NO_3_)_2_·6H_2_O, Ni(NO_3_)_2_·6H_2_O and C_4_H_6_N_2_ as precursors, an amount of Zn(NO_3_)_2_·6H_2_O and Ni(NO_3_)_2_·6H_2_O (the corresponding ratios of Zn/Ni equal to 1 : 9, 3 : 7, 5 : 5, 7 : 3, 9 : 1) were dissolved into 20 mL distilled water. The mole number of C_4_H_6_N_2_ was 10 times larger than that of the sum molar number of Zn(NO_3_)_2_·6H_2_O and Ni(NO_3_)_2_·6H_2_O, these precursors were dissolved into 10 mL distilled water. Thereafter, the C_4_H_6_N_2_ solution was added dropwise into the above mixture solution while stirring. The final mixture was stirred for 24 h at room temperature. The precipitates were rinsed with deionized water and anhydrous ethanol several times, and then dried in a vacuum oven at 60 °C for 12 h. The ZIF-8 and ZnCo-MOF could be obtained by replacing the Ni(NO_3_)_2_·6H_2_O with Zn(NO_3_)_2_·6H_2_O and Co(NO_3_)_2_·6H_2_O, respectively.

### Synthesis of the g-C_3_N_4_–ZnM-ZIFs and g-C_3_N_4_–MoS_2_–ZnM-ZIFs

**Fig. 1:**
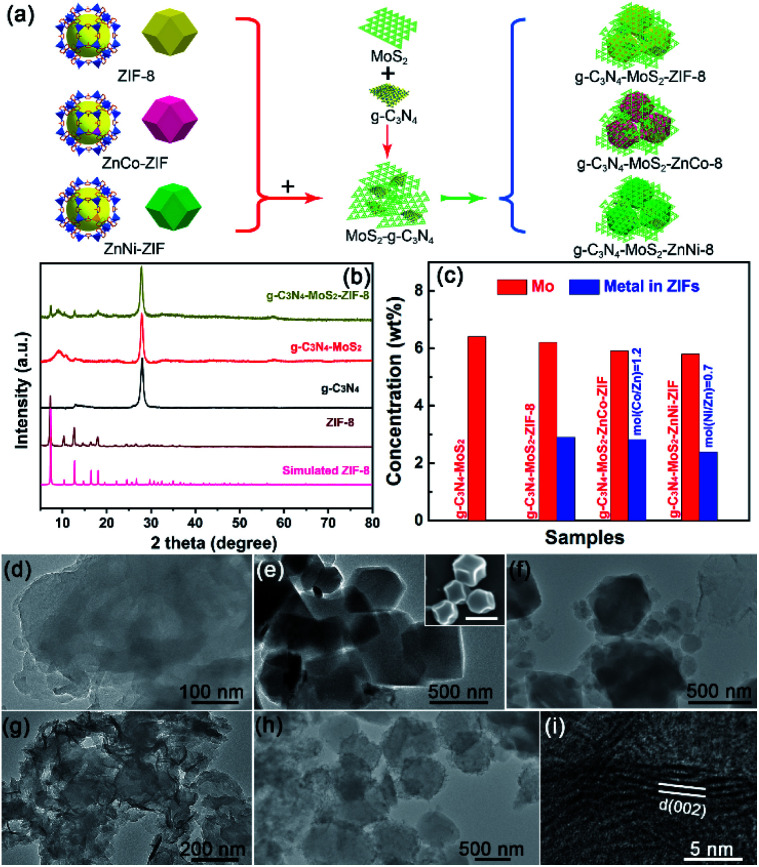
(a) Scheme illustrating the synthesis of the ternary g-C_3_N_4_–MoS_2_–ZnNi-ZIF heterostructure. (b) PXRD patterns of the g-C_3_N_4_, g-C_3_N_4_–MoS_2_, g-C_3_N_4_–MoS_2_-ZIFs and ZIF-8 (c) Mo and metals concentrations in the g-C_3_N_4_–MoS_2_ and g-C_3_N_4_–MoS_2_-ZIFs from ICP spectroscope. TEM images of the (d) g-C_3_N_4_, (e) ZnNi-ZIF (inset showing the SEM image), (f) g-C_3_N_4_–ZnNi-ZIF, (g) g-C_3_N_4_–MoS_2_, (h) g-C_3_N_4_–MoS_2_–ZnNi-ZIF, and (i) HRTEM image of the g-C_3_N_4_–MoS_2_–ZnNi-ZIF.

Suitable amount of Zn(NO_3_)_2_·6H_2_O and Ni(NO_3_)_2_·6H_2_O (the corresponding ratios of Zn/Ni equal to 5 : 5) and as-prepared g-C_3_N_4_ were added into 20 mL distilled water. C_4_H_6_N_2_ with 10 mole ratios of the amount of Zn(NO_3_)_2_·6H_2_O and Ni(NO_3_)_2_·6H_2_O were dissolved into 10 mL distilled water. Thereafter, the C_4_H_6_N_2_ solution was added dropwise into the above mixture solution while stirring. The final mixture was stirred for 24 h at room temperature. The precipitates were rinsed with deionized water and anhydrous ethanol several times, and then dried in a vacuum oven at 60 °C for 12 h. The g-C_3_N_4_–ZIF-8 and g-C_3_N_4_–ZnCo-ZIF could be obtained by replacing the Ni(NO_3_)_2_·6H_2_O by Co(NO_3_)_2_·6H_2_O. The g-C_3_N_4_–MoS_2_–ZnM-ZIFs were prepared using the similar method with g-C_3_N_4_–MoS_2_ as precursor as shown in Fig. 1a.

### Photocatalytic hydrogen production

The photocatalytic H_2_-production experiments were performed *via* photocatalytic H_2_-production activity evaluation system (CEL-SPH2N, CEAU-Light, China) in a 300 mL Pyrex flask, and the flask openings were sealed with silicone rubber septum. A 300 W xenon arc lamp was used as all light source, and was positioned 13 cm away from the reaction solution to trigger the photocatalytic reaction. A 300 W xenon arc lamp through a UV-cutoff filter (*λ* = 420–800 nm) was used as a visible light source, and was positioned 13 cm away from the reaction solution to trigger the photocatalytic reaction. FZ-A visible-light radiometer (made in the photoelectric instrument factory of Beijing Normal University, China) was used to measure the focused intensity on the flask (*i.e.*, 200 mW cm^−2^). In a typical photocatalytic H_2_-production experiment, 20 mg of the as-prepared photocatalyst was suspended in 50 mL of mixed aqueous solution of 5 mL C_6_H_15_NO_3_ (AR, 98%). To ensure the anaerobic condition in the reactor before irradiation, the system was vacuumed for 5 min by the use of vacuum pump for the entire removal of dissolved oxygen. The photocatalyst particles were suspended throughout the experiments by employing a continuous magnetic stirrer at the bottom of the reactor. H_2_ content was analyzed by gas chromatography (GC-7900, CEAU-Light, China). All glassware were carefully rinsed with DI water prior to usage. The photocatalytic stability was also performed in the same processing parameters. Every 12 h the sacrificial regent was renewed for the evaluation of the photocatalytic stability in vacuum.

## Results and discussion

The powder X-ray diffraction patterns (PXRD) of g-C_3_N_4_ with MoS_2_ and/or ZnM-ZIF is demonstrated in Fig. 1b. The characteristic diffraction peaks of g-C_3_N_4_–MoS_2_-ZIFs could be clearly observed. For comparison, g-C_3_N_4_-based samples with and without MoS_2_ is shown in Fig. 1b, the diffraction peak of the MoS_2_ (around 9°) shifted to lower theta values (around 1°) due to the larger interplanar spacing, verifying the successful fabrication of ternary g-C_3_N_4_–MoS_2_–ZnM-ZIF heterostructure as shown in Fig. 1b and S1,† and has been previously reported.^50^ Notably, PXRD pattern justified the successful g-C_3_N_4_–ZnM-ZIF–MoS_2_ heterostructure formation at first and then reduction of peak shows that material after hetero-structure composite is 2D material which is amorphous in nature. Furthermore, Fourier Transform Infrared Spectroscopy (FTIR) spectra were further employed for confirming the formation of g-C_3_N_4_–ZnM-ZIF heterostructures. As shown in Fig. S2a,† the typical peak of ZnNi-ZIF appeared at 422 cm^−1^ belonged to the Ni–N stretching vibration, the peaks at 2960 and 2925 cm^−1^ could be attributed to the asymmetric absorption vibration of the C–H bond in the –CH_3_ group, the absorption peaks at 748 cm^−1^ was caused by stretching vibration of the C=N bond in 2-methylimidazole, and the intense absorption peaks at 1000 cm^−1^ were derived from the plane bending vibration of the imidazole ring.^51^ Distinctly, the characteristic FTIR peaks of ZnM-ZIF and g-C_3_N_4_ could also be clearly observed in the g-C_3_N_4_–MoS_2_–ZIF-8 (CNMZ) and g-C_3_N_4_–MoS_2_–ZnCo-ZIF (CNMZC) samples (Fig. S2b†), verifying the existence of ZnM-ZIF in the g-C_3_N_4_–MoS_2_–ZnM-ZIF. The concentrations of Mo and the metals in ZnM-ZIFs in the g-C_3_N_4_–MoS_2_ with and without ZnM-ZIFs were investigated *via* inductively coupled plasma (ICP) spectroscope (Fig. 1c). The Mo content in the relevant samples was very close to the theory predicted value. Whereas, the metals content in the ZnM-ZIFs was lower than the given value (10 wt%), which may be attributed to the underreaction between the metals and ligand. Moreover, the molar ratio of the Zn/Co and Zn/Ni was close the supposed value, which indicates that the synthesized bimetallic ZIF meets expectations.

Transmission electron microscopy (TEM) was employed for investigating the morphology of the g-C_3_N_4_ embedded with ZnM-ZIF and/or MoS_2_. The dense nanosheet morphology of pristine g-C_3_N_4_ could be observed in Fig. 1d, and compared with ZIF-8 and ZnNi-ZIFs, the as-prepared ZnNi-ZIF exhibited regular truncated rhombic dodecahedron structure as confirmed by the TEM and scanning electron microscope (SEM) images (Fig. 1e and S3a–c†). For the CNZN, the microstructure of g-C_3_N_4_ nanosheet enwrapped MOF blocks is clearly observable, proved the formation of CNZN surface heterostructure (Fig. 1f), which was further confirmed by the SEM images of g-C_3_N_4_–ZnM-ZIFs as depicted in Fig. S3d–f.† The CNM was a 2D–2D surface heterostructure (Fig. 1g) as reported in the previous publication.^50^ Notably, the CNM nanosheet was distributed uniformly over the surface of ZnNi-ZIF as demonstrated in Fig. 1h, and the (002) lattice plane of MoS_2_ could be clearly observed from CNMZN (Fig. 1i), which has an enlarged interlayer spacing (0.95 nm). Moreover, the g-C_3_N_4_–MoS_2_–ZnM-ZIFs exhibited similar morphology on SEM images (Fig. S3g–i†). Further adopted the elemental mapping to investigate the distribution of different components in the ternary CNMZN (Fig. S4†). The result confirmed that all the C, N, Zn, Ni, S and Mo uniformly dispersed. Further verifying both the MoS_2_ and g-C_3_N_4_ distributed evenly on the skeleton of ZnNi-ZIF.

**Fig. 2:**
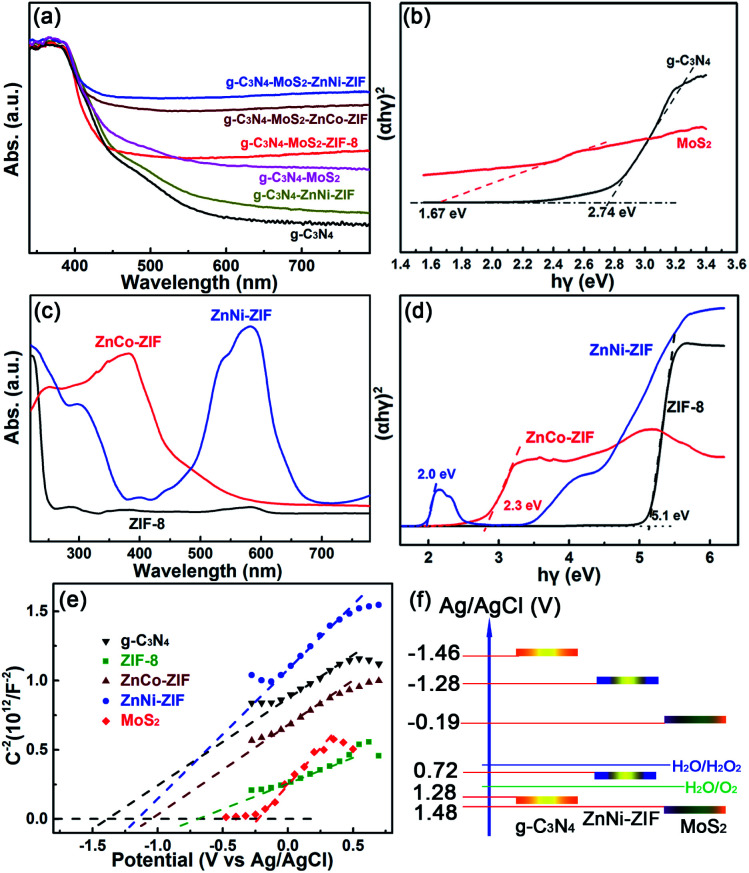
The UV-Vis absorption spectra and (*αhν*)^2^*versus hν* curves of (a and b) g-C_3_N_4_ loaded with MoS_2_ and/or ZnM-ZIF, and (c and d) ZnM-ZIFs. The (e) Mott–Schottky plots of the g-C_3_N_4_, MoS_2_ and the ZnM-ZIF, and (f) the band structure matching in the ternary g-C_3_N_4_–MoS_2_–ZnNi-ZIF heterostructure.

The visible light absorption and the bandgap of the as-prepared g-C_3_N_4_-based heterostructures were investigated *via* the UV-Vis diffuse absorption spectra (Fig. 2a). After embedding ZnM-ZIFs, the absorption of the g-C_3_N_4_ in the visible light region has a little enhancement (Fig. S5†), while for the g-C_3_N_4_–MoS_2_, the absorption rate is significantly improved (Fig. S6†). It is worth noting that the co-loading of the MoS_2_ and ZnM-ZIF exhibited a synergistic effect for the absorption of visible light, and the absorption intensity in the visible light region was in the order of CNMZC > CNMZN > ZNMZ. The bandgaps of g-C_3_N_4_ and MoS_2_ calculated according to the Kubelka–Munk (KM) method are 2.74 and 1.67 eV (Fig. 2b).^52,53^ As displayed in Fig. 2c, simultaneous doping of Co and Ni could increase the visible light absorption capacity of ZIF-8, as further confirmed by the bandgaps for ZIF-8, ZnCo-ZIF and ZnNi-ZIF of 5.1, 2.3 and 2.0, respectively (Fig. 2d). To determine the band structure matching and charge separation mechanism among g-C_3_N_4_, MoS_2_, and ZnM-ZIFs, the Mott–Schottky plot measurements were used to investigate the flat band potential of them. As shown in Fig. 2e, the ZIF-8, ZnCo-ZIF, and ZnNi-ZIF exhibited flat-band potentials of −0.89, −1.24 and −1.28 V *vs.* Ag/AgCl, respectively, which were lower than that of the pristine g-C_3_N_4_ (−1.46 V), while higher than that of MoS_2_ (−0.19 V). Therefore, the band structure matching in the ternary g-C_3_N_4_–MoS_2_–ZnNi-ZIF heterostructure could be inferred in Fig. 2f. This band structure indicated the transfer of photo-generated electron from the conduction band (CB) of g-C_3_N_4_ and ZnNi-ZIFs to that of MoS_2_. On the other hand, the holes were transferred into the valence band (VB) of ZnNi-ZIF, where the H_2_O_2_ was preferentially formed efficiently owing to the highly porous morphology of ZnNi-ZIF. The band structure matching of the g-C_3_N_4_–ZnNi–MoS_2_ not only facilitated the separation and utilization efficiency, but also improved the visible light absorption between g-C_3_N_4_, ZnNi-ZIF and MoS_2_.

**Fig. 3:**
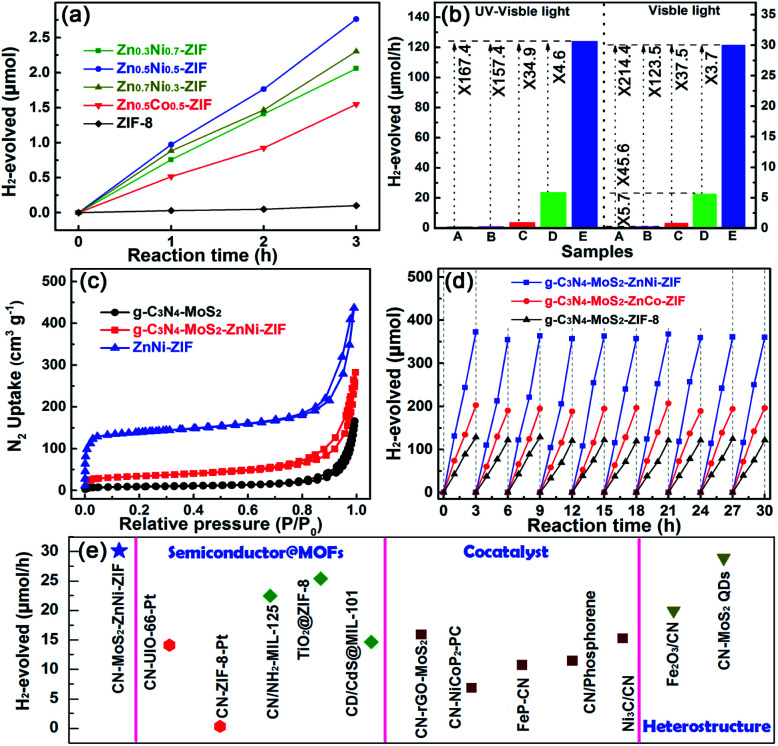
The photocatalytic H_2_-production activities of (a) ZnM-ZIF with different molar ratio of Zn to metals. The comparison results of (b) the photocatalytic hydrogen production activity of the pristine (A) g-C_3_N_4_, (B) ZnNi-ZIF, (C) g-C_3_N_4_–ZnNi-ZIF, (D) g-C_3_N_4_–MoS_2_ and (E) ternary g-C_3_N_4_–MoS_2_–ZnNi-ZIF under UV-Vis and visible light irradiation. (c) Nitrogen adsorption desorption curve of g-C_3_N_4_, g-C_3_N_4_–MoS_2_, ZnNi-ZIF and the optimal ternary heterostructure. (d) Photocatalytic H_2_-production stability of the ternary g-C_3_N_4_–MoS_2_–ZnM-ZIFs. (e) Comparison results of the photocatalytic activity of the g-C_3_N_4_–MoS_2_–ZnNi-ZIF with the previously reported g-C_3_N_4_ (CN) and MOFs-based heterostructures.

Notably, in sharply contrast to the negligible photocatalytic hydrogen production activity of ZIF-8 because of its large bandgap (5.1 eV), the bare bimetallic ZnM-ZIFs exhibited sizable increase in hydrogen evolution activity as displayed in Fig. 3a. The optimal photocatalytic HER performance occurs in the Zn_0.5_Ni_0.5_-ZIF, achieved 0.92 μmol h^−1^ under UV-Vis light irradiation, which could be comparable to that of g-C_3_N_4_ (0.71 μmol h^−1^). Moreover, the photocatalytic HER performance of Zn_0.5_Co_0.5_-ZIF slightly lower than that of the g-C_3_N_4_, which was 0.51 μmol h^−1^, which. The photocatalytic HER performance of the CNZN with different weight ratio of ZnNi-ZIF (Zn : Ni = 1 : 1) were compared in Fig. S7,† the optimal HER performance was obtained in the heterostructure sample containing 10 wt% ZnNi-ZIF. Notably, the further adoption of the MoS_2_ (optimal dosage of 6 wt%, Fig. S8†) cocatalyst remarkably improved the photocatalytic HER activity under UV-Vis (Fig. S9a†) and visible light irradiation (Fig. S9b†).

In order to better evaluate the important role of the MoS_2_ as well as the ZnM-ZIF, the photocatalytic hydrogen production activity is improved after embedded g-C_3_N_4_ with MoS_2_ and then with ZnM-ZIF. The systematic comparison of photocatalytic hydrogen production activity g-C_3_N_4_, g-C_3_N_4_–MoS_2_, ZnM-ZIF is showed in Fig. 3b and S10.† Under UV-Vis light irradiation, the pristine g-C_3_N_4_ and ZnNi-ZIF exhibited very limited hydrogen production rate of only 0.71 and 0.92 μmol h^−1^, respectively, and the adoption of MoS_2_ and ZnNi-ZIF both dramatically increased the hydrogen production rate of g-C_3_N_4_ due to the utilization and transport efficiency improving of the charge carriers, respectively. Co-embedding of ZnNi-ZIF and MoS_2_ enhanced the photocatalytic activity of 124.02 μmol h^−1^, which was 167.4 times larger than that of the pristine g-C_3_N_4_, and was 34.9 and 4.6-fold of that of the g-C_3_N_4_ singly loaded with ZnNi-ZIF and MoS_2_, respectively. Hence, the photocatalytic hydrogen production results proved that the embedding of ZnNi-ZIF and MoS_2_ showed obvious synergistic effect for increasing the photocatalytic HER activity. Specifically, the CNZN and the CNM exhibited photocatalytic HER performance of 5.7 and 64.2 times larger than that of pristine g-C_3_N_4_, indicating the ZnNi-ZIF and MoS_2_ played an important role on improving the number of exposed active sites and utilization efficiency of the charge carriers, respectively. It was worth noting that the photocatalytic hydrogen production rate of ternary CNMZN, CNMZC and CNMZ was 3.7, 1.9 and 1.2-fold in comparison with sum of photocatalytic hydrogen production of CNM and the corresponding g-C_3_N_4_–ZnM-ZIFs (Fig. S10†), which confirmed the synergistic effect of the embedding of MoS_2_ and ZnM-ZIF and facilitate the transportation and utilization efficiency of photo-generated charge carriers.

The large specific surface area, caused by improved number of exposed active sites, was also an important element for the high photocatalytic activity of CNMZN,^5,54^ and the nitrogen adsorption/desorption isotherms (Fig. 3c) was employed to reveal the vital function of ZnNi-ZIF on increasing the porosity of the ternary heterostructure. Adopt MoS_2_ could increase the specific surface area (*S*_BET_) of pure g-C_3_N_4_ to a certain extent. More obviously, CNMZN exhibited much larger nitrogen uptake and *S*_BET_ of 142.73 m^2^ g^−1^ than that of CNM (33.89 m^2^ g^−1^) owing to the highly porous ZnNi-ZIF (501.34 m^2^ g^−1^). In addition to the excellent photocatalytic hydrogen production activity, the CNMZN and ZNMZC exhibited very good photocatalytic stability after 30 h continuous irradiation (Fig. 3d). In comparison with the pristine ZnM-ZIFs and their heterostructures, the characteristic FTIR peaks of ZnM-ZIF could also be clearly observed in the ZnNi-ZIF and CNMZN heterostructure after the photocatalytic reaction, which further indicated the excellent photocatalytic stability of the ternary CNMZN heterostructure (Fig. S11a†), and the unchanged FTIR spectra of CNMZ as well as CNMZC were also displayed in Fig. S11b.† To further verify the improved transport, separation and utilization efficiency of the photo-generated charge carrier in g-C_3_N_4_ caused by the adoption of MoS_2_ and ZnM-ZIFs, the photocatalytic hydrogen production performance of the optimal CNMZN (30.1 μmol h^−1^) was compared with the photocatalytic hydrogen production activity of g-C_3_N_4_, or MOF-based photocatalysts in previous publication under visible light irradiation. As displayed in Fig. 3e, the optimal CNMZN exhibited very competitive photocatalytic activity in comparison with those of semiconductor-MOFs heterostructures,^2,14,35,55–57^ g-C_3_N_4_-loaded with different types of cocatalysts,^9–11,22,23,54^ and g-C_3_N_4_-based heterostructures.^9,58^

**Fig. 4:**
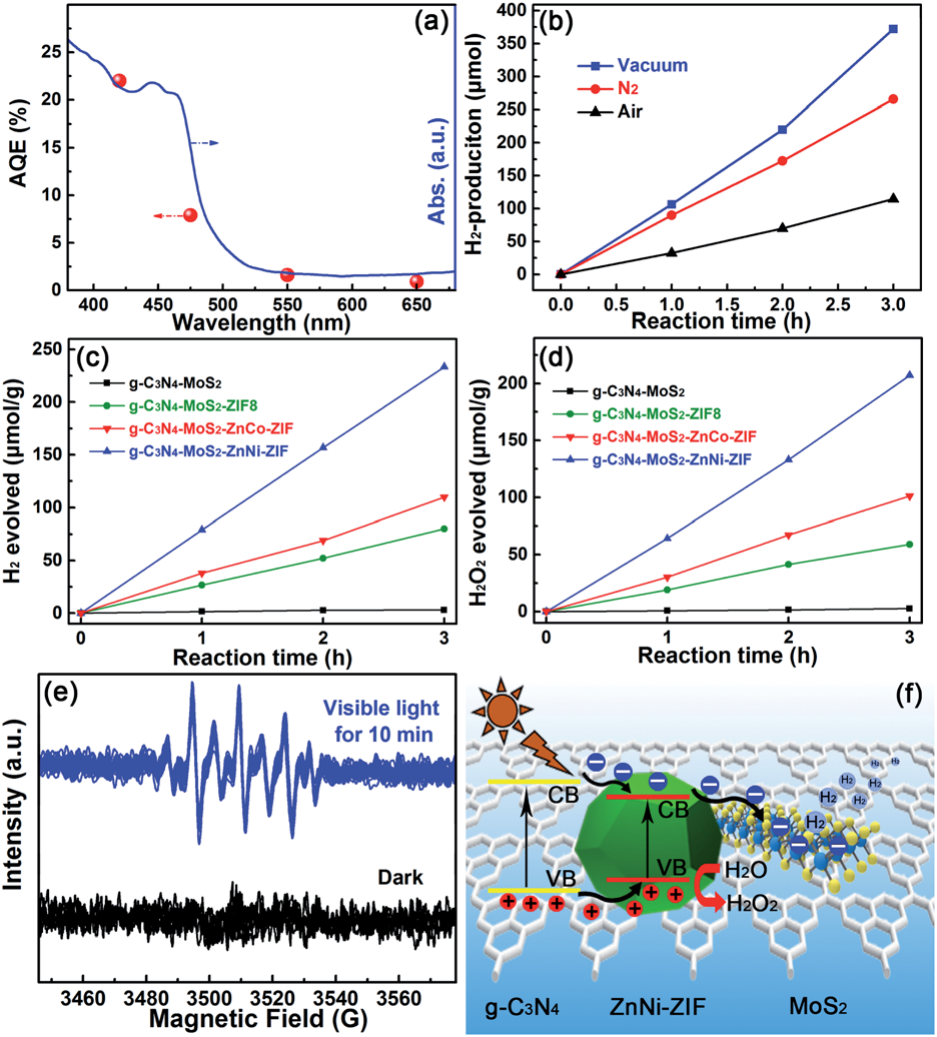
(a) Wavelength dependence of the apparent quantum efficiencies for the ternary g-C_3_N_4_–MoS_2_–ZnNi-ZIF heterostructures. (b) Photocatalytic H_2_-evolution rate of the optimal g-C_3_N_4_–MoS_2_–ZnNi-ZIF under different atmosphere in TEOA mixture solution. (c) Photocatalytic hydrogen and (d) H_2_O_2_ production activity of the g-C_3_N_4_–MoS_2_–ZnNi-ZIF in pure water under UV-Vis light irradiation. (e) EPR responses of the DMPO–OH spin adduct in g-C_3_N_4_–MoS_2_–ZnNi-ZIF. (f) Possible separation and utilization mechanism of the photo-generated charge carriers in the optimal g-C_3_N_4_–MoS_2_–ZnNi-ZIF heterostructure.

The optimal g-C_3_N_4_–MoS_2_–ZnNi-ZIF heterostructure exhibited an excellent apparent quantum yield (AQY) value of 22.0% at 420 nm. The AQY and photocatalytic hydrogen production rate of optimal g-C_3_N_4_–MoS_2_–ZnNi-ZIF under different specific wavelength illuminations was evaluated and exhibited (Fig. 4a and Table S1†) with the irradiation wavelengths of 420, 475, 550 and 650 (±8) nm, the HER values 18.18, 9.22, 2.76 and 1.42 μmol h^−1^ (Table S1†), respectively, which indicated the HER of the optimal g-C_3_N_4_–MoS_2_–ZnNi-ZIF (CNMZN) photocatalyst was associated with its UV-visible diffuse reflection spectra, and the electron-promoted photocatalytic hydrogen evolution reaction. Different wavelengths further assured the photocatalytic activity of the optimal g-C_3_N_4_–MoS_2_–ZnNi-ZIF heterostructure. The considerable photocatalytic hydrogen production in optimal g-C_3_N_4_–MoS_2_–ZnNi-ZIF even with the irradiation wavelengths of 650 (±8) nm also verified that g-C_3_N_4_–MoS_2_ not only acted as the promoter of the charge carriers and high-performance catalyst, but also facilitated the absorption capacity of visible light. These results are consistent with the DRS and PL results of the g-C_3_N_4_–MoS_2_ and ZnNi-ZIF-based heterostructure.

Under UV-Vis light irradiation the photocatalytic hydrogen production of the optimal ternary CNMZN heterostructure was evaluated at different atmosphere for comprehensively assessing its practical applications value (Fig. 4b). The photocatalytic HER activity of the optimal ternary heterostructure under N_2_ atmosphere decreased slightly due to the easier hydrogen release under vacuum. It should be noted that even in air atmosphere with oxygen facilitating the backward reaction,^13^ the optimal CNMZN exhibited considerably photocatalytic hydrogen evolution activity, this result revealed that the CNMZN ternary heterostructure was suitable for practical applications.

Impressively, as demonstrated in Fig. 4c, sharp contrast to the very weak hydrogen evolution of the g-C_3_N_4_–MoS_2_ without sacrificial agent (pure water), the CNMZ, CNMZC and CNMZN ternary heterostructures exhibited considerable H_2_-evolution performance of 26.7, 36.6 and 77.8 μmol h^−1^ g^−1^ under UV-Vis light irradiation with the considerable H_2_O_2_ production rate (Fig. 4d). For deeply investigate the formation process of H_2_O_2_, using 5,5-dimethyl-1-pyrroline N-oxide (DMPO) as the spin trapping agent, Electron Paramagnetic Resonance (EPR) responses were adopted to test the oxidation products during the photocatalytic reaction in pure water of CNMZN. As exhibited in Fig. 4e, the characteristic signals of –OH could be observed in the DMPO aqueous solution after the visible light irradiation, indicating the formation of –OH through singe electron oxidation of water. On the base of the above-mentioned band structure and photocatalytic activity results, the mechanism of action of photo-exited charge carriers in the CNMZN photocatalyst was proposed in Fig. 4f.

**Fig. 5:**
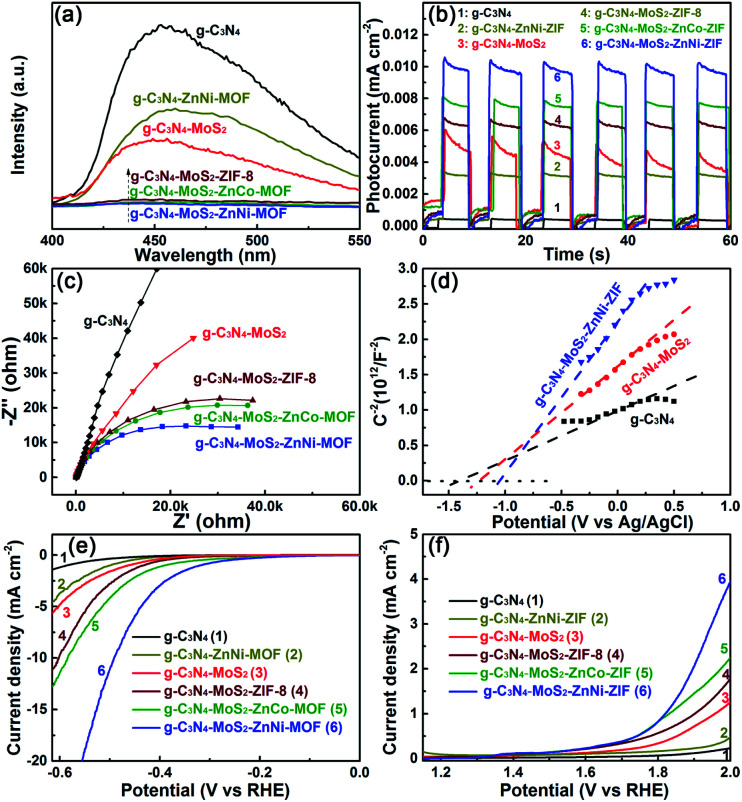
Effect of different type ZnM-ZIF and the adoption of MoS_2_ on (a) the PL emission spectra, (b) transient photocurrent responses, (c) EIS spectra, (d) the Mott–Schottky plots, and electrocatalytic (e) HER and (f) OER response of the g-C_3_N_4_-based heterostructures.

The photo-induced electrons transferred from the CB of g-C_3_N_4_ and ZnNi-ZIFs to that of MoS_2_, being beneficial for the photocatalytic hydrogen production owing to the low HER overpotential and large current density of MoS_2_. Simultaneously, the photo-exited holes were transferred into the VB of ZnNi-ZIF, where the holes could be timely consumed by sacrificial agent due to the highly porous morphology of the ZnNi-ZIF. For investigating the important role of ZnM-ZIF and/or MoS_2_ on suppressing the recombination of the photo-generated electron–hole pairs of the g-C_3_N_4_ based heterostructure, the room temperature photoluminescence (PL) spectra excited at 360 nm (Fig. 5a) was employed. In contrast to the very strong PL peak of the pristine g-C_3_N_4_ (around 450 nm), the dramatically quenched PL signals were observed against the g-C_3_N_4_–ZnM-ZIF (M = Co, Cu, Ni) and CNM (Fig. S12†), owing to the increased porosity and separation as well as utilization efficiency of the charge carriers, respectively and the relative order of PL peaks for g-C_3_N_4_–ZnM-ZIF samples was found to be; g-C_3_N_4_ > g-C_3_N_4_–ZnCo > g-C_3_N_4_–ZnCu > g-C_3_N_4_–ZnNi. Notably, the ternary samples, co-embedded with ZnM-ZIF and MoS_2_, showed very weak PL peaks, and the relative order of PL response intensity for the samples with different types of ZnM-ZIF was found to be: CNMZ > CNMZC > CNMZN, which was consistent with the trend of the photocatalytic hydrogen evolution activity.

The g-C_3_N_4_ based photocatalysts were investigated through the transient photocurrent and electrochemical impedance spectroscopy (EIS) measurements to explore the transport and excitation behavior of the photo-exited charge carriers. As shown in Fig. 5b, with the repeated on/off cycles of UV-Vis light irradiation, all the g-C_3_N_4_ based samples exhibited the steady and reproducible photocurrent responses. The rapid recombination of charge carriers made the photocurrent density of the pristine g-C_3_N_4_ very weak. Both the g-C_3_N_4_–ZnM-ZIF (Fig. S13†) and CNM demonstrated stronger photocurrent response owing to the increased specific surface area and separation as well rapid consumed electrons, respectively. The relative order for the photocurrent density for the g-C_3_N_4_–ZnM-ZIF samples was found to be; g-C_3_N_4_ > g-C_3_N_4_–ZnCo > g-C_3_N_4_–ZnCu > g-C_3_N_4_–ZnNi, which showed that after incorporation of ZnM-ZIF (M = Cu, Co, Ni) the photocurrent density increase that was consistent with photocatalytic HER activity. Impressively, for the ternary heterostructure, the co-loading of ZnM-ZIF and MoS_2_ exhibited the synergistic effect for facilitating the photocurrent response, and the value of the photo-current for the ternary heterostructures with different type of ZnM-ZIF was consistent with the photocatalytic HER activity.

The EIS results of the g-C_3_N_4_, ZnM-ZIF g-C_3_N_4_–ZnM-ZIF and g-C_3_N_4_–MoS_2_–ZnM-ZIF were exhibited in Fig. 5c, the ternary g-C_3_N_4_–MoS_2_–ZnM-ZIF displayed much smaller semicircle radius than that of and pristine g-C_3_N_4_, ZnNi-ZIF, g-C_3_N_4_–ZnCo-ZIF and g-C_3_N_4_–ZnCu-ZIF (Fig. S14†), indicating that the interfacial transfer of the charge carriers was dramatically enhanced as verified by the smaller arc radius, which is normally the evidence of a lower electron transfer resistance. Notably, the CNMZN owning the optimal photocatalytic HER performance showed the smallest arc radius. In addition, the hydrogen evolution behaviors were evaluated by the Mott–Schottky plots and electrocatalytic HER and OER measurements. As depicted in Fig. 5d, the CNM displayed a more negative flat-band potentials than that of the pristine g-C_3_N_4_, indicating its improved reduction capacity. Notably, after the embedding of ZnM-ZIF, the sample exhibited higher reduction capacity as confirmed by their further upward shift in the flat-band potential, and the CNMZN with the optimal photocatalytic activity possessed the most negative flat-band potential. It was extensively reported that the cocatalyst with low electrocatalytic HER and/or oxygen evolution reaction (OER) overpotential and high current density could boost the utilization efficiency of charge carriers, thereby improve the photocatalytic activity. Moreover, the HER and OER cocatalyst could play a synergistic effect by co-loading to enhance the photocatalytic activity.^59^ The HER and OER capacity of the g-C_3_N_4_-based heterostructure with MoS_2_ and/or ZnM-ZIF were evaluated using HER and OER measurements. As could be observed from Fig. 5e and f, the embedding of both MoS_2_ and ZnNi-ZIF could dramatically promote the electrocatalytic HER and OER performance, and the co-embedding of MoS_2_ and ZnM-ZIF over g-C_3_N_4_ further facilitated the electrocatalytic HER and OER performance, and the ternary CNMZN displayed the smallest HER and OER overpotential, and the largest current density, being consistent with the photocatalytic HER performance of the g-C_3_N_4_–MoS_2_–ZnM-ZIFs.

## Conclusions

Herein, we have successfully designed and fabricated various bimetallic zeolite imidazole framework (ZnM-ZIF, M = Co, Ni) as the photocatalytic hydrogen production catalyst with considerable photocatalytic HER activity. Furthermore, photocatalytic HER activity of zeolite imidazole framework (ZnM-ZIF, M = Co, Ni) was enhanced by the introduction of g-C_3_N_4_–MoS_2_ 2D–2D heterostructure. g-C_3_N_4_–MoS_2_ 2D–2D heterostructure facilitate the separation as well as utilization efficiency of the photo-generated charge carriers due to the lower HER flat-band potential of MoS_2_. ZnM-MOF not only inhibited the aggregation of the CNM heterostructure, but also further improved the separation and transport efficiency of the charge carriers in the ternary g-C_3_N_4_–MoS_2_–ZnM-ZIF. Consequently, the optimal g-C_3_N_4_–MoS_2_–ZnNi-ZIF exhibited the extraordinary photocatalytic HER activity, which is 214.4, 37.5, and 3.7 times larger than that of the pristine g-C_3_N_4_, g-C_3_N_4_–ZnNi-ZIF and g-C_3_N_4_–MoS_2_, respectively, and exhibited a H_2_-evolution performance of 77.8 μmol h^−1^ g^−1^ under UV-Vis light irradiation coupled with oxidation of H_2_O into H_2_O_2_. This work would furnish an innovative path for MOF candidate and open a new method for the better utilization of porous MOF-based heterostructure for photocatalyst.

## Conflicts of interest

There are no conflicts to declare.

## Supplementary Material

RA-011-D1RA00781E-s001
