# Supplementary material for: Bimetallic zeolite-imidazole framework-based heterostructure with enhanced photocatalytic hydrogen production activity
Source: RSC Adv. 2021 Mar 1;11(16):9048–56. doi: 10.1039/d1ra00781e (PMC8695345; doi:10.1039/d1ra00781e)
Supplement: RA-011-D1RA00781E-s001 [file RA-011-D1RA00781E-s001.pdf]

Electronic Supplementary Material (ESI) for RSC Advances.
This journal is © The Royal Society of Chemistry 2021

Supporting Information

Bimetallic Zeolite-imidazole framework-based heterostructure with enhanced photocatalytic hydrogen production activity

Nayab Arif, Ye-Zhan Lin, Kai Wang, Yi-Chuan Dou, Yu Zhang, Kui Li, * Shiquan Liu* and Fu-Tian Liu

School of Materials Science and Engineering, University of Jinan, Jinan 250022, China.

Email: mse_lik@ujn.edu.cn; vctrliu@hotmail.com

4. Supporting Figures

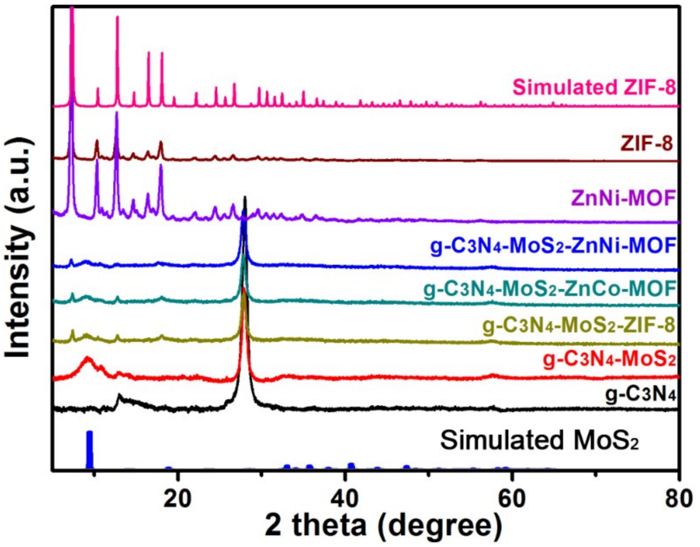

Fig. S1. PXRD patterns of the g-C_3N_4-MoS_2, ZnM-ZIFs and g-C_3N_4-MoS_2-ZnM-ZIFs heterostructures

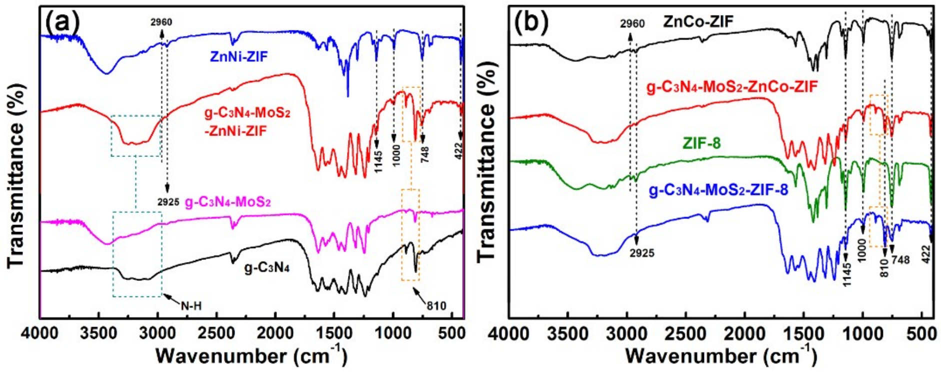

Fig. S2. The (a) FTIR analysis of the ZnNi-ZIF and its heterostructure, (b) ZIF-8, ZnCo-ZIF and their heterostructures.

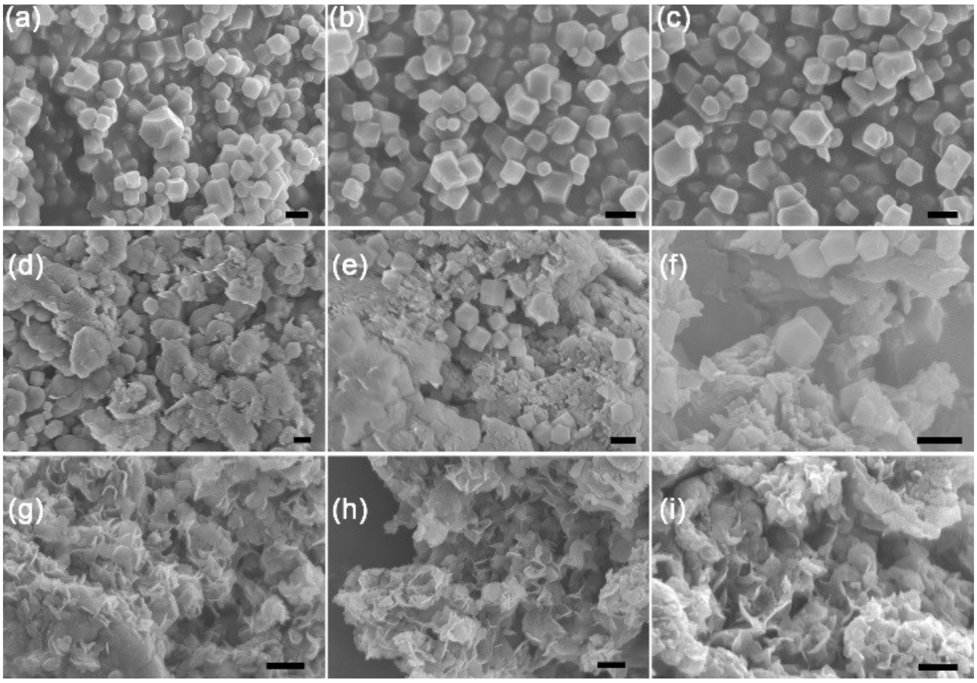

Fig. S3. The SEM images (Scale bar of 500 nm) of (a) ZIF-8, (b) ZnCo-ZIF, (c) ZnNi-ZIF, (d) g-C_3N_4-ZIF-8, (e) g-C_3N_4-ZnCo-ZIF, (f) g-C_3N_4-ZnNi-ZIF, (g) g-C_3N_4-MoS_2-ZIF-8, (h) g-C_3N_4-MoS_2-ZnCo-ZIF and (i) g-C_3N_4-MoS_2-ZnNi-ZIF.

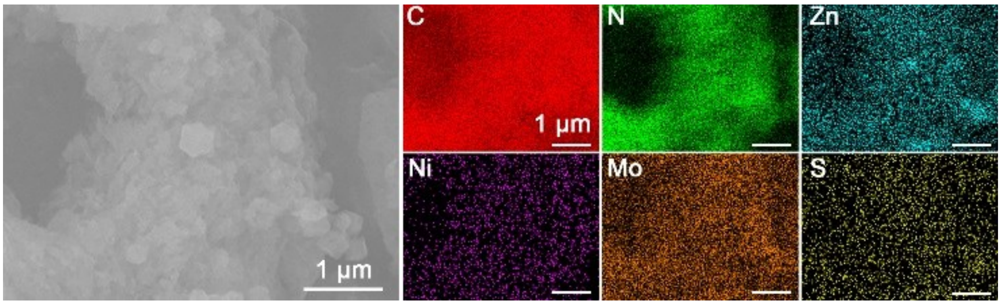

Fig. S4. The elemental mapping of the g-C_3N_4-MoS_2-ZnNi-ZIF.

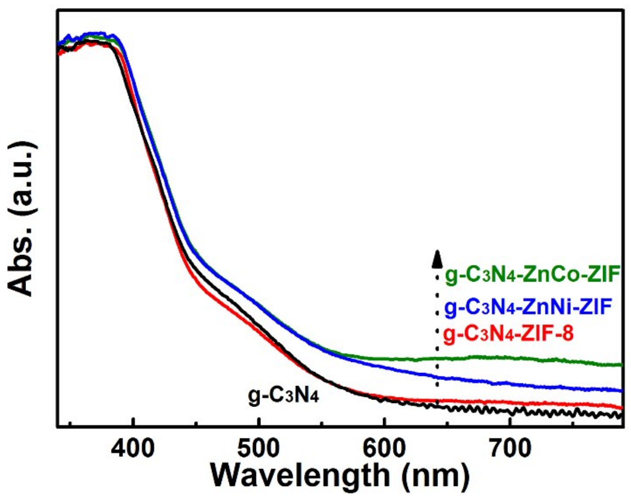

Fig. S5. The UV-vis diffuse reflection spectra of g-C_3N_4 and g-C_3N_4-ZnM-ZIF heterostructures.

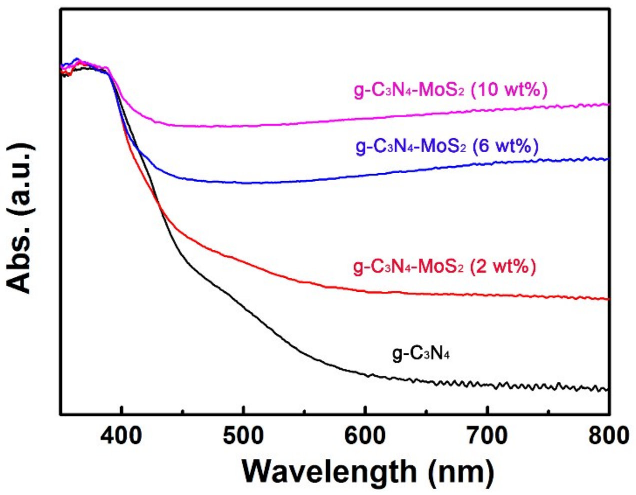

Fig. S6. The UV-vis diffuse reflection spectra of g-C_3N_4-MoS_2 heterostructures.

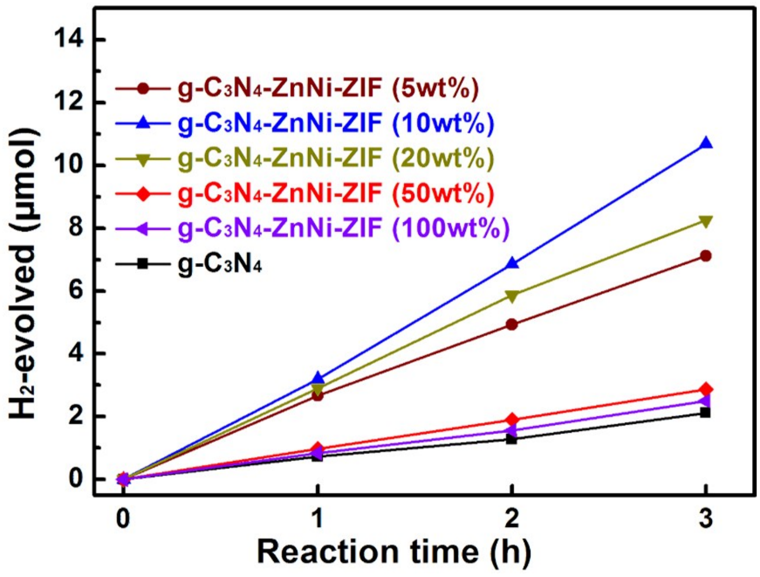

Fig. S7. The photocatalytic H_2-production activities of g-C_3N_4-ZnNi-ZIF with different content of ZnNi-ZIF UV-visible light irradiation.

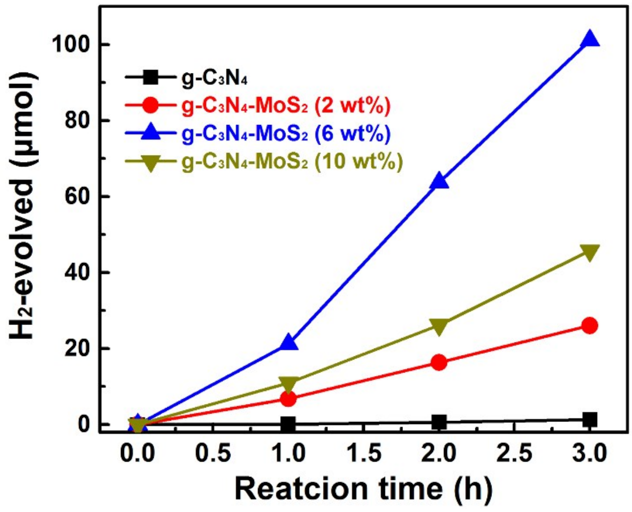

Fig. S8. Time dependent of photocatalytic hydrogen production activity of g-C_3N_4-MoS_2 heterostructures.

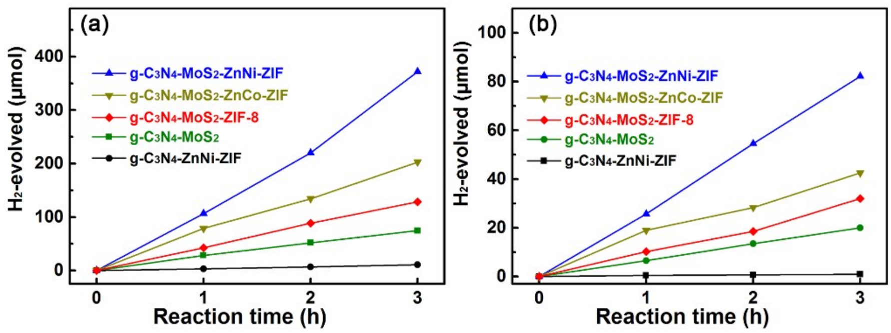

Fig. S9. Effect of the adoption of MoS_2 and g-C_3N_4 on the photocatalytic activity of ZnM-ZIFs under (a) UV-visible and (b) visible light irradiation.

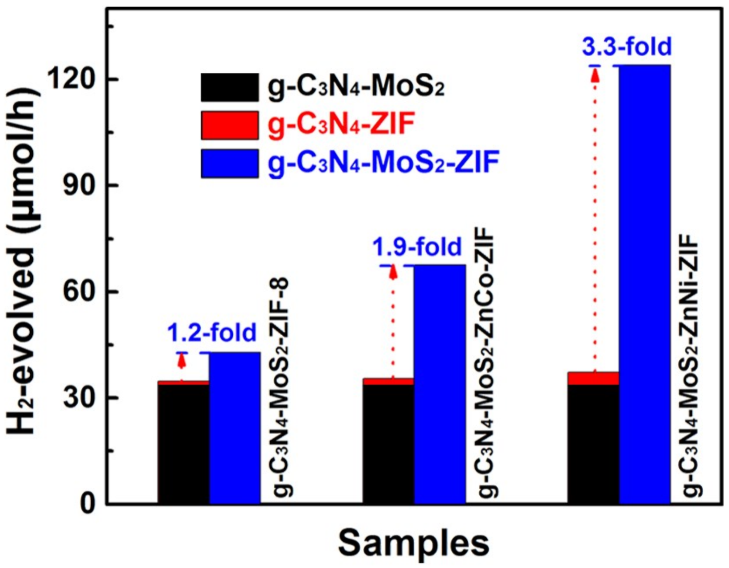

Fig. S10. The comparison results sum photocatalytic activity of g-C_3N_4-ZIFs and g-C_3N_4-MoS_2 with that of ternary g-C_3N_4-MoS_2-ZnM-ZIFs under UV-vis light irradiation.

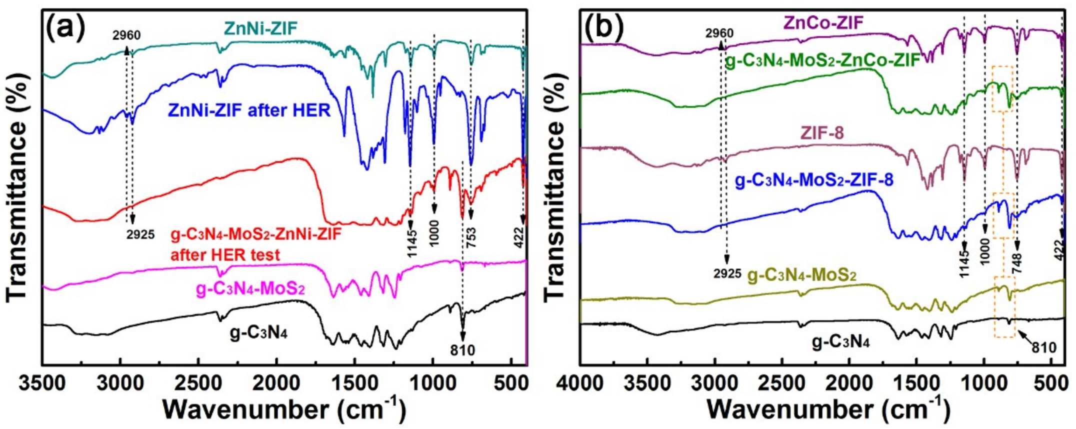

Fig. S11. The FTIR analysis of the (a) ZnNi-ZIF-based and (b) ZIF-8 as well as ZnCo-ZIF-based heterostructures after the photocatalytic hydrogen production reaction.

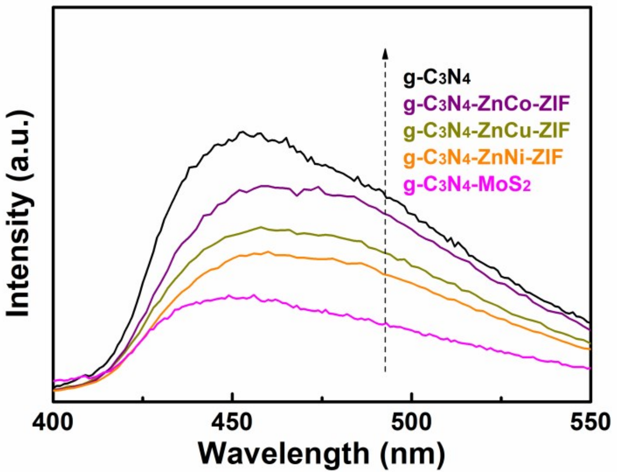

Fig. S12. The room temperature photoluminescence (PL) spectra of g-C_3N_4, g-C_3N_4-MoS_2 and g-C_3N_4-ZnM-ZIF heterostructures.

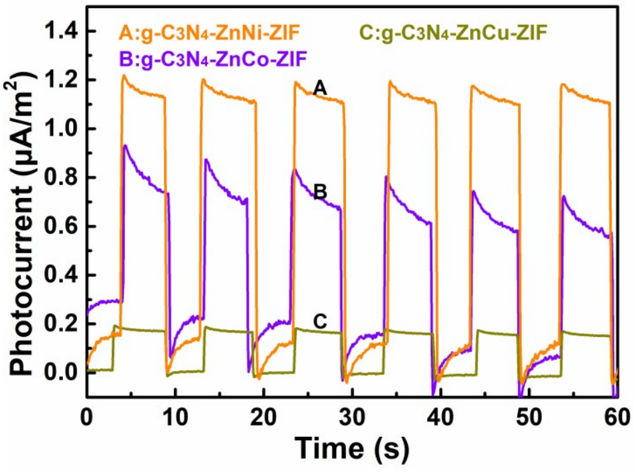

Fig. S13. The transient photocurrent responses of g-C_3N_4 and g-C_3N_4-ZnM-ZIF heterostructures.

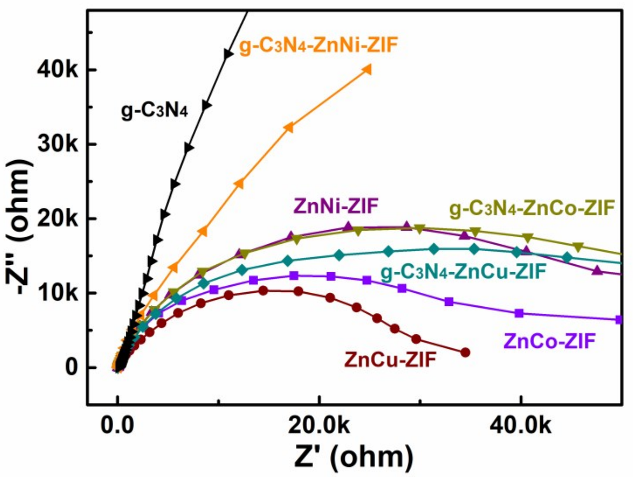

Fig. S14. The electrochemical impedance spectroscopy (EIS) spectra of g-C_3N_4, ZnM-ZIF, and g-C_3N_4-ZnM-ZIF heterostructures.

5. Supporting Table

Table S1. Calculated apparent quantum efficiency (AQE) of g-C_3N_4-MoS_2-ZnNi-ZIF at different wavelengths.

Wavelength (nm)	H_2 Evolved (μmol)	Light Intensity (mW)	AQEs (%)
420	18.18	13.1	22.0
475	9.22	18.4	7.9
550	2.76	20.3	1.6
650	1.42	16.5	0.9

λ=420 nm

$$N = \frac{E\lambda}{hc} = \frac{13.1 \times 10^{-3} \times 3600 \times 420 \times 10^{-9}}{6.626 \times 10^{-34} \times 3 \times 10^{8}} = 9.96 \times 10^{19}$$

$$AQE = \frac{the\ number\ of\ reacted\ electrons}{the\ number\ of\ incident\ photons} \times 100\%$$

$$= \frac{2 \times the\ number\ of\ evolved\ H_2\ molecules}{N} \times 100\%$$

$$= \frac{2 \times 6.02 \times 10^{23} \times 18.18 \times 10^{-6}}{9.96 \times 10^{19}} = 22.0\%$$

λ=475 nm

$$N = \frac{E\lambda}{hc} = \frac{18.4 \times 10^{-3} \times 3600 \times 475 \times 10^{-9}}{6.626 \times 10^{-34} \times 3 \times 10^{8}} = 1.58 \times 10^{20}$$

$$AQE = \frac{the\ number\ of\ reacted\ electrons}{the\ number\ of\ incident\ photons} \times 100\%$$

$$= \frac{2 \times the\ number\ of\ evolved\ H_2\ molecules}{N} \times 100\%$$

$$= \frac{2 \times 6.02 \times 10^{23} \times 9.22 \times 10^{-6}}{1.58 \times 10^{20}} = 7.9\%$$

λ=550 nm

$$N = \frac{E\lambda}{hc} = \frac{20.3 \times 10^{-3} \times 3600 \times 550 \times 10^{-9}}{6.626 \times 10^{-34} \times 3 \times 10^{8}} = 2.02 \times 10^{20}$$

$$AQE = \frac{the\ number\ of\ reacted\ electrons}{the\ number\ of\ incident\ photons} \times 100\%$$

$$= \frac{2 \times the\ number\ of\ evolved\ H_2\ molecules}{N} \times 100\%$$

$$= \frac{2 \times 6.02 \times 10^{23} \times 2.76 \times 10^{-6}}{2.02 \times 10^{20}} = 1.6\%$$

λ=650 nm

$$N = \frac{E\lambda}{hc} = \frac{16.5 \times 10^{-3} \times 3600 \times 650 \times 10^{-9}}{6.626 \times 10^{-34} \times 3 \times 10^{8}} = 1.94 \times 10^{20}$$

$$AQE = \frac{the\ number\ of\ reacted\ electrons}{the\ number\ of\ incident\ photons} \times 100\%$$

$$= \frac{2 \times the\ number\ of\ evolved\ H_2\ molecules}{N} \times 100\%$$

$$= \frac{2 \times 6.02 \times 10^{23} \times 1.42 \times 10^{-6}}{1.94 \times 10^{20}} = 0.9\%$$
